# Effectiveness of a health literacy intervention targeting both chronic kidney disease patients and health care professionals in primary and secondary care: a quasi-experimental study

**DOI:** 10.1007/s40620-024-02058-8

**Published:** 2024-10-04

**Authors:** Marco D. Boonstra, Matheus S. Gurgel do Amaral, Gerjan Navis, Mariken E. Stegmann, Ralf Westerhuis, Josue Almansa, Andrea F. de Winter, Sijmen A. Reijneveld

**Affiliations:** 1 Department of Health Sciences, University Medical Center Groningen, Hanzeplein 1, 9713GZ Groningen, The Netherlands; 2 Department of Nephrology, University Medical Center Groningen, Groningen, The Netherlands; 3 Department of General Practice and Elderly Care Medicine, University Medical Center Groningen, Groningen, The Netherlands

**Keywords:** Health literacy, Chronic kidney disease, Self-management, Communication

## Abstract

**Background:**

Chronic kidney disease (CKD) patients with limited health literacy are at risk for faster disease progression. To counteract this problem, we developed ‘Grip on your Kidneys’ (GoYK), an intervention targeting patients and health care professionals. We assessed the effect on self-management, patient activation, clinical parameters, consultation quality, and the professionals’ use of health literacy strategies. We further evaluated the process.

**Methods:**

A quasi-experimental study included 147 patients with CKD and 48 professionals from Dutch general practices and nephrology clinics. Patients and professionals in the intervention group (IG) received GoYK. Control patients received care-as-usual from the participating professionals. Data were collected with questionnaires and from patient records at baseline (*T*0), 4 months (*T*1) and 9 months (*T*2).

**Results:**

No effects on self-management and patient activation were found. Conversely, at *T*2, the proportion of patients with hypertension decreased in the intervention group (odds ratio = 0.45, 95% confidence interval (95%CI) [0.20, 0.99]). In the intervention group, more lifestyle topics were discussed, at *T*1 (difference = 0.80, 95%CI [0.28, 1.31]) and *T*2 (difference = 0.69, 95%CI [0.14, 1.25]). Furthermore, several outcomes related to consultation quality improved. Professionals in the intervention group improved the use of health literacy strategies more, at T1 (difference = 0.64, 95%CI [0.33, 0.95]) and *T*2 (difference = 0.56, 95%CI [0.19, 0.93]). In general, patients and professionals considered GoYK to be useful.

**Conclusions:**

GoYK is promising, and offers a blueprint to optimize care for patients with limited health literacy. Researchers should develop and test interventions like GoYK, focusing on patients at risk for CKD, and with very low health literacy.

**Graphical abstract:**

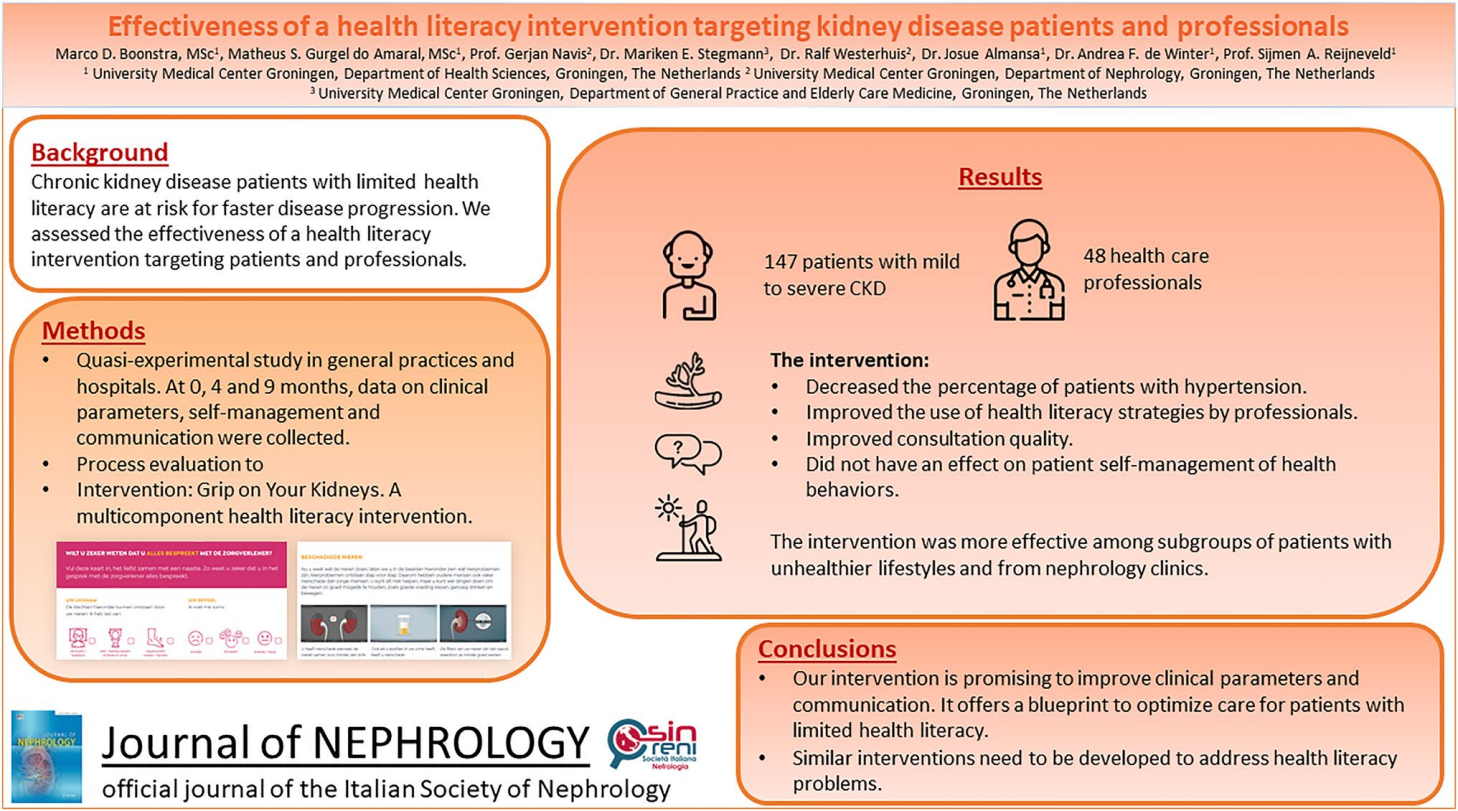

**Supplementary Information:**

The online version contains supplementary material available at 10.1007/s40620-024-02058-8.

## Introduction

Approximately 25% of chronic kidney disease (CKD) patients have limited health literacy [1], a risk factor for CKD onset and progression [2]. Health literacy is the capacity to obtain, process, and understand basic health information and services, needed to make appropriate health decisions [3]. CKD patients with limited health literacy often have problems in self-management [4]. Health literacy problems also lead to suboptimal communication between patients and health care professionals [5]. Intervening on these factors is central in this study.

Better self-management is associated with better health in CKD patients [6]. Self-management is the ability to manage symptoms, treatment, physical and psychosocial consequences, and lifestyle changes inherent to living with a chronic condition [7]. For CKD patients, integrating health behaviors, such as eating less salt, is central in their self-management. An important precondition for self-management is patient activation, defined as having the knowledge, skills, and confidence to manage health [8].

Regularly, professionals fail to activate the patients and to adequately support self-management [9]. They overestimate patients, use difficult language, or do not meet the specific needs of patients with limited health literacy [10]. Patients with limited health literacy struggle to participate in consultations and often have lower understanding of treatment recommendations [11]. The above illustrates the need to optimize self-management and communication between patients and professionals.

Health literacy interventions have been effective in improving the competences of patients [12] and professionals [13], but are uncommon in CKD care. Most interventions target patients with severe CKD or kidney failure, and are not fit to prevent kidney decline in earlier CKD stages. Evidence for their effectiveness is weak and interventions targeting professionals in CKD settings do not exist [14]. To overcome this gap, we developed and pilot-tested Grip on Your Kidneys (GoYK) [15].

GoYK aligns with theory from the European project entitled Intervention Research on Health Literacy among the Ageing Population (IROHLA) [16]. The intervention aims to activate patients by improving their knowledge on CKD and self-management, to motivate patients to integrate healthy behaviors into their lives and to help them prepare for consultations. GoYK also supports patients to maintain healthier behaviors in the long term and to teach professionals to tailor communication to the needs of patients with limited health literacy [15].

We evaluated the effectiveness of GoYK, compared to care-as-usual. We assessed the effects on self-management of health behaviors, patient activation, clinical parameters, quality of consultations, and on professionals’ use of health literacy communication strategies. Additionally, we assessed the use and usefulness of, and satisfaction with, the intervention.

## Methods

The CONSORT checklist guided the reporting of this study. This study was registered in the ‘Overview of Medical Research in the Netherlands’ (nr: 23167) and approved by the Medical Ethical Committee of the University Medical Center Groningen (nr: 201900534).

### Design and setting

We conducted a two-armed, non-blinded, quasi-experimental study within general practices and nephrology clinics in the Netherlands. Most Dutch residents are registered with a general practitioner, who usually treats patients with mild to moderate CKD. Patients with more severe CKD are referred to nephrology clinics.

### Participants and sampling

Two practices and three clinics provided GoYK, whereas two practices and two clinics provided care-as-usual. We recruited patients with mild to severe CKD (CKD stages 2–4) and professionals from December 2020 until September 2021. Eligible patients were ≥18 years, had ≥3 months of CKD based on estimated glomerular filtration rate (eGFR), and had regular consultations with participating professionals. Patients with kidney failure and major cognitive or life-threatening conditions, and professionals with previous health literacy training were excluded.

Professionals were approached by email and selected eligible patients from their electronic patients records. Four hundred thirty patients received an information letter and consent form during consultations or at their home addresses. At variance with the registered trial, we included patients of various health literacy levels, but oversampled patients with limited health literacy. This enabled to analyze the intervention effectiveness among health literacy subgroups, and to guarantee sufficient participation, during COVID-19.

### Sample size

To achieve 80% power, an a priori calculation for multilevel analysis set our needed sample at 91 patients nested under 38 professionals in each group. First, the number of professionals was based upon the outcome ‘use of health literacy communication strategies’ and assumed an effect size of 0.65. Second, we estimated the number of patients needed to detect a change of 10.0 in the intervention-control group difference for the patient activation measure [8] with a standard deviation of 15.8 [17], and a two-tailed alpha of 0.05. To allow for 5% attrition among professionals and 10% attrition among patients, we aimed to enroll 40 professionals and 102 patients per group.

### Intervention

GoYK is a multi-component intervention, offered on top of care-as-usual, developed in co-creation with patients and professionals [15]. For patients, the intervention enhances CKD knowledge, communication and self-management through simple text and visuals. Professionals followed an e-learning course and attended a lesson session on health literacy, based upon the effective health literacy training of Kaper et al. [18]. Supplementary file 1 contains a detailed description.

### Care-as-usual

In line with guidelines [19], patients had two to four annual consultations with a general practitioner, nephrologist or a specialized nurse practitioner. If needed, patients in the nephrology clinics received additional care from a nurse practitioner, dietitian or social worker. Patients in CKD-stage 4 received more intensive care, often having consultations every four to six weeks. Protocolled consultation topics in all settings were lab results, medication, lifestyle and, if applicable, co-morbid diseases.

### Procedures

For patients and professionals, we collected data at baseline (*T*0), and after 4 (*T*1) and 9 months (*T*2). We used paper and online questionnaires, sent to the participants’ e-mail or home address. Data collection was tailored to limited health literacy patients, for example by allowing help with the questionnaires. Reminders were sent if questionnaires were not returned, after one and two weeks. Professionals helped to collect data on the patients’ clinical parameters from the electronic patient records.

### Randomization and blinding

Within-organization randomization was infeasible. Blinding was not possible due to intervention visibility. Patient groups were concealed until study start to prevent bias.

### Measures

Below are the primary and secondary outcomes. Supplementary file 2 provides an overview of the references upon which these outcomes are based.

#### Primary outcomes for patients

***Self-management of health behaviors; Salt intake*** – Item adopted from Humalda et al. (2020). We asked how many days a week patients consumed salty foods within nine different food groups. ***Alcohol**** –* We asked how many days per week patients drank alcohol and the average number of doses on those days. ***Physical activity**** –* We asked the number of days a week patients did at least 30 min of physical activity, based upon O’Halloran et al. (2020). We dichotomized into adequate (5–7 days) and inadequate (0–4 days). ***Fluid intake*** – We asked about the number of millilitres of fluid intake per day. We dichotomized it into inadequate (< 1500 mL) and adequate (≥1500 mL). ***Medication adherence*** – The Medication Adherence Report Scale of Chan et al. (2020), 5 items, was used. A total score between 5 and 25 was calculated. ***Patient activation*** –Measured with the Patient Activation Measure (PAM-13). The PAM-13 gives a score between 0–100 [8].

#### Secondary outcomes for patients

***Health literacy*** – 10 items of the All Aspects of Health Literacy Scale (AAHLS) by Chinn et al. (2013). The AAHLS provides a total score between 10 and 30. Patients had limited health literacy when they scored ≤ 25. ***Quality of the consultation*** – Based on the communication framework of Haes and Bensing (2009), we developed four statements with 5-point Likert scales asking if patients felt understood, could ask questions and share emotions, and if professionals listened well. For each statement, we calculated the percentage of patients agreeing as a measure for quality. A fifth item asked to select topics discussed during a consultation. We calculated the number of lifestyle topics. ***CKD clinical parameters*** –We obtained eGFR, blood pressures and BMI. Patients with a systolic blood pressure ≥ 140 or diastolic blood pressure ≥ 90 were classified as hypertensive.

#### Outcomes for professionals

The primary outcome for professionals was ***self-reported use of health literacy communication strategies***. We asked 31 questions within domains: gathering information, providing information, shared decision-making and enabling self-management. Each item contained 7-point Likert scales (never-always). Sixteen items were from Kaper et al. (2018). We added fifteen items, in line with our intervention objectives. The secondary outcome was ***health literacy knowledge.*** We asked 6 questions with 7-point Likert scales from Kaper et al. on the professionals’ knowledge regarding health literacy (2018).

#### Background characteristics

For patients, we collected data on sex, age, education, marital status, years of CKD, smoking, and co-morbidities. For professionals, data included sex, age, profession, health setting, experience, expected contact with limited health literacy patients, and communication training.

### Process evaluation

In the intervention group, we asked patients about the use (yes/no) and the usefulness of (useful, neutral, not useful), and the satisfaction with (yes/no, and grading 1–10) GoYK. Professionals answered questions on usefulness and satisfaction, and statements with 1–7 Likert scales, to check if they expected to use the learned strategies in the future.

### Analyses

First, missing patient data were imputed (% missing = 0.6–10.6%) by performing 20 imputations using fully conditional specification, and predictive mean matching for continuous variables. The model included baseline variables associated with the outcomes to improve the prediction. For professional outcomes, we did not perform imputation because missing seemed not at random, as four of the seven non-returned questionnaires came from physicians. Second, we determined whether a two-level structure was present, with patients nested under professionals, by assessing the intra-class correlations at the professional level. These were close to zero, meaning that one- and two-level analyses yielded similar results. We reported the one-level analyses.

We calculated baseline descriptive statistics and evaluated differences between the intervention group and care-as-usual. Then, we assessed the intervention effect by performing linear or multinomial logistic regressions, with significance set at *p* < 0.05. We adjusted the patient analyses for baseline eGFR given a difference between the intervention group and care-as-usual. As a sensitivity analysis, we reran patient analyses with the non-imputed dataset and without correction for eGFR. We performed subgroup analyses for (1) patients at risk (i.e. with inadequate health behaviors, hypertension and perceiving the consultation quality as low), (2) patients with limited health literacy, (3) patients from general practices and nephrology clinics, and (4) patients who used ≥2 intervention components. In the process evaluation, we calculated descriptive statistics for all outcomes and analyzed differences between patients with limited and adequate health literacy, and from general practices and clinics.

## Results

### Participant flow

**Fig. 1:**
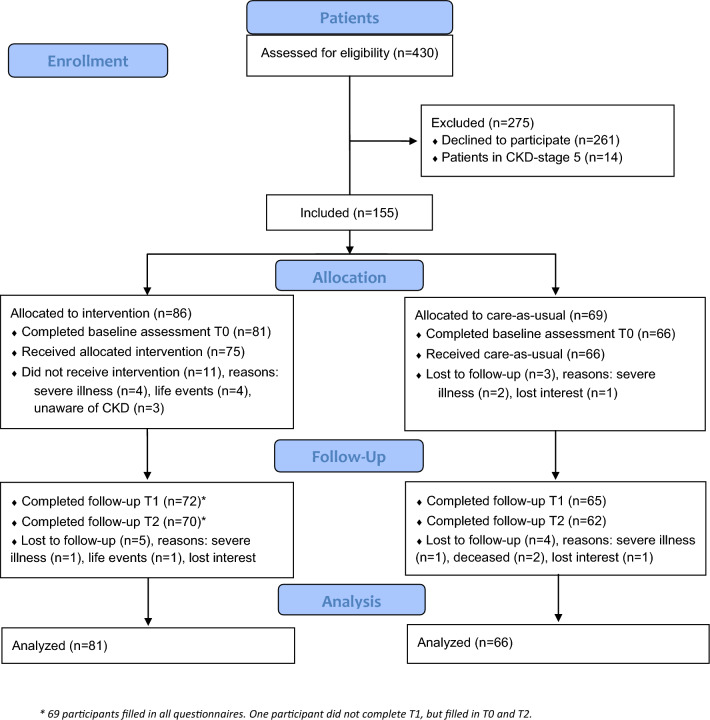

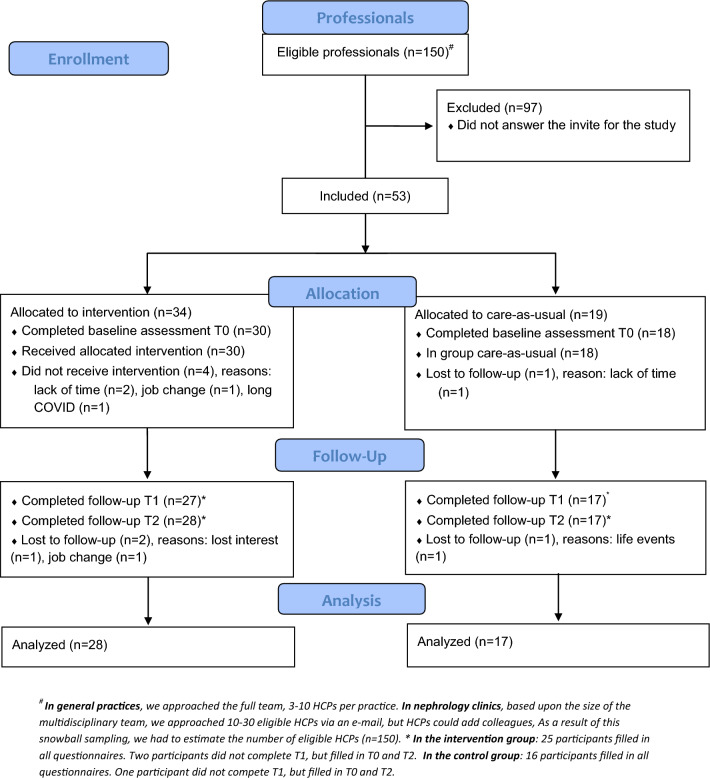
Flowcharts presenting enrollment, allocation, follow-up and analysis of the patients and professionals in our study

Flowcharts are shown in Fig. 1. Among patients, 169 (39%) consented and 155 were included, 86 in the intervention and 69 in care-as-usual groups. Fifty-three professionals (35%) were included, 48 filled in the baseline measure and 45 were in the final analyses.

### Participant background

Baseline characteristics in the two groups were similar (Table 1). Patients in the control group more often had severe CKD (*p* = 0.004) and their mean eGFR was lower (*p* = 0.001) compared to the intervention group. Among patients in the intervention group, 64.2 percent had limited health literacy, comparable to the 75.8 percent in care-as-usual. For smoking and alcohol intake, baseline measures indicated a healthy sample, compared to the general Dutch population (see supplementary file 1).

**Table 1:** Background characteristics of patients and professionals involved in the study, by group

Patients (*n* = 147)	CAU (*n* = 66)	Intervention (*n* = 81)	*p*-value	*n*
Age, median (IQR)	75.0 (11.8)	73.7 (14.8)	0.127^a^	147
Male sex, %	63.6	61.7	0.812^b^	147
Health literacy, median (IQR)	23.0 (3.5)	24.0 (5.0)	0.100^a^	147
Limited health literacy, %	75.8	64.2	0.130^b^	147
Educational level, %				
*High*	33.3	33.3	0.137^b^	147
*Intermediate*	21.2	34.6		
*Low*	45.5	32.1		
Partnered, %	83.3	77.8	0.725^b^	147
Health setting, %				
*Nephrology clinic*	59.1	59.3	0.984^b^	147
*General practice*	40.9	40.7		
Smoke, %	10.6	8.6	0.687^b^	147
Drink alcohol, %	50.0	53.1	0.710^b^	147
eGFR, median (IQR)	32.0 (28.0)	44.0 (23.0)	**0.001**^a^	138
CKD stage, %				
*2*	0.0	13.5	**0.004**^c^	138
*3*	54.0	63.5		
*4*	46.0	23.0		
Years of CKD, %				
*< 1 year*	7.8	11.7	0.613^b^	142
*1–3 years*	18.8	24.7		
*3–5 years*	18.8	18.2		
*5–10 years*	23.4	14.3		
*> 10 years*	31.2	31.1		
Reported multimorbidity, *%*				
*At least one comorbidity*	97.0	88.9	0.064^b^	147
*Diabetes*	30.3	22.2		
*Hypertension*	65.2	51.9		
*Cardiovascular disease*	24.2	19.8		
*Hypercholesterolemia*	16.7	12.3		
*Lung disease*	19.7	4.9		
*Depression / anxiety*	9.1	7.4		
*Rheumatologic*	31.8	25.9		
*Other diseases*	28.8	29.6		

Health care professionals (*n* = 48)	CAU (*n* = 18)	Intervention (*n* = 30)	*p*-value	*N*
Age^#^, median (IQR)	49.5(16)	47.5 (14)	0.110^a^	48
Male sex, %	27.8	20.0	0.724^d^	48
Health setting, %				
*Hospital care*	72.2	76.7	0.743^d^	48
*General practice*	27.8	23.3		
Profession, %				
*General practitioner*	11.1	6.7	0.908^d^	48
*GP nurse practitioner*	5.6	10.0		
*Nephrologist*	27.8	23.3		
*Hospital nurse practitioner*	5.6	20.0		
* Nurse*	16.7	13.3		
*Dietitian*	11.1	13.3		
*Social worker*	11.1	6.7		
*Other*	11.1	6.7		
Years of experience, %				
*0–2 years*	5.6	10.0	0.812^d^	48
*2–5 years*	22.2	20.0		
*5–10 years*	16.7	16.7		
*10–15 years*	5.6	13.3		
*15–20 years*	16.7	23.3		
*Over 20 years*	33.3	16.7		
Contact with people with limited health literacy, %				
*Now and then*	27.8	16.7	0.449^d^	48
*Regularly*	55.6	73.3		
*Very often*	16.7	10.0		
Received general communication training during career, %				
*0–2 times*	16.7	30.0	0.689^d^	48
*3–4 times*	33.3	26.7		
*5 times or more often*	50.0	43.3		

Descriptive statistics based on the crude dataset. Numbers in bold indicate significant results

*CAU* care-as-usual, *n* sample size, *IQR* interquartile range, *eGFR* estimated glomerular filtration rate, *CKD* chronic kidney disease

^a^Mann-Whitney test

^b^Pearson’s Chi-square

^c^Pearson’s Chi-square with CKD stages 2–3 merged to overcome analysis problems resulting from lacking patients in CKD-stage 2 in CAU

^d^Fisher exact test, due to *N* =  ≤ 5 in a subgroup

### Patient outcomes

There were no significant effects on the primary outcomes of self-management and patient activation. For the secondary outcomes, we found significant effects (see Table 2). At *T*2, the intervention group showed a 14.2% decrease in the percentage of patients with hypertension, while in care-as-usual there was a 3.5% increase (OR = 0.45, 95%CI [0.20, 0.99]). The number of lifestyle topics discussed during consultations in the intervention group improved at *T*1 (*B* = 0.80, 95%CI [0.28, 1.31] and T2 (*B* = 0.69, 95%CI [0.14–1.25]). At *T*1, the intervention group showed improved outcomes related to the quality of the consultation. For example, 11.8% more intervention group patients agreed that the professional listened to their preferred approach, compared to 4.6% fewer in care-as-usual (OR = 2.61, 95%CI [1.05, 6.47]).

**Table 2:** Linear and logistic regressions of primary and secondary patient outcomes for the change between T0-T1 and T0-T2, corrected for eGFR

Continuous primary outcomes	Group	Baseline	*T*1	*T*2	Intervention effect T0-T1		Intervention effect T0-T2	
					*B* (95% CI)	*p*	*B* (95% CI)	*p*
PAM^1^ mean (SD)	CAU	55.6 (10.0)	56.3 (14.2)	59.0 (13.1)	0.25 ( – 5.41 to 5.92)	0.930	– 3.90 (-8.25 to 0.46)	0.080
	IG	61.7 (13.6)	63.1 (17.5)	62.5 (11.4)				
Medication adherence^2^ mean (SD)	CAU	23.8 (2.0)	23.7 (2.5)	23.6 (2.1)	– 0.08 ( – 1.11 to 0.95)	0.882	– 0.05 (-1.05 to 0.96)	0.930
	IG	23.4 (2.2)	23.2 (2.9)	23.2 (2.6)				
Alcohol (doses per week) mean (SD)	CAU	6.4 (5.5)	5.7 (5.0)	6.3 (6.3)	0.20 ( – 1.25 to 1.64)	0.790	– 1.07 ( – 2.84 to 0.70)	0.236
	IG	4.6 (6.5)	4.2 (5.1)	3.7 (5.1)				
Salt intake (days per week) mean (SD)	CAU	5.1 (1.9)	4.8 (2.1)	5.1 (2.1)	– 0.03 ( – 0.68 to 0.63)	0.939	– 0.59 ( – 1.40 to 0.22)	0.153
	IG	4.9 (1.9)	4.5 (2.0)	4.4 (2.0)				

Dichotomous primary outcomes	Group	Baseline	*T*1	*T*2	OR (95% CI)	*p*	OR (95% CI)	*p*
Adequate physical activity^3^*%*	CAU	44.1	37.5	42.4	1.95 (0.78 to 4.84)	0.152	0.98 (0.43 to 2.22)	0.962
	IG	34.6	42.1	35.7				
Fluid intake of ≥ 1.5L a day *%*	CAU	62.8	60.9	66.8	1.25 (0.56 to 2.82)	0.588	0.87 (0.37 to 2.03)	0.748
	IG	48.0	58.3	56.4				

Continuous secondary outcomes	Group	Baseline	*T*1	*T*2	*B* (95% CI)	*p*	*B* (95% CI)	*p*
Health literacy^4^ *mean (SD)*	CAU	23.0 (3.1)	23.7 (3.2)	23.6 (3.3)	– 0.33 ( – 1.52 to 0.87)	0.593	– 0.27 ( – 1.53 to 0.99)	0.674
	IG	23.8 (3.1)	24.0 (3.1)	24.0 (3.4)				
N of lifestyle topics discussed in consultation mean (SD)	CAU	1.5 (1.1)	1.0 (1.2)	1.3 (1.3)	**0.80 (0.28 to 1.31)**	**0.003**	**0.69 (0.14 to 1.25)**	**0.014**
	IG	1.1 (1.3)	1.4 (1.4)	1.5 (1.5)				
eGFR^5^ mean (SD)	CAU	33.9 (14.9)		32.7 (15.0)			– 2.39 ( – 6.01 to 1.23)	0.195
	IG	43.3 (16.2)		39.7 (15.4)				
BMI mean (SD)	CAU	28.1 (5.4)	28.3 (5.6)	27.9 (4.9)	0.23 ( – 1.41 to 1.87)	0.779	0.31 ( – 1.23 to 1.86)	0.690
	IG	27.5 (4.4)	27.9 (5.9)	27.6 (5.1)				

Dichotomous secondary outcomes	Group	Baseline	*T*1	*T*2	OR (95% CI)	*p*	OR (95% CI)	*p*
Hypertension *%*	CAU	46.9	44.2	50.4	0.58 (0.25 to 1.33)	0.194	**0.45 (0.20 to 0.99)**	**0.047**
	IG	42.1	29.3	27.9				
Feeling understood by professionals *%*	CAU	70.5	63.5	61.7	2.66 (0.95 to 7.48)	0.063	1.29 (0.59 to 2.81)	0.519
	IG	79.9	80.7	67.0				
Encouraged by professionals to ask questions *%*	CAU	53.6	44.9	52.4	**2.38 (1.04 to 5.45)**	**0.040**	1.05 (0.48 to 2.32)	0.898
	IG	61.4	62.3	55.7				
Professionals listen to my preferred approach *%*	CAU	58.3	52.7	55.6	**2.61 (1.05 to 6.47)**	**0.038**	1.06 (0.50 to 2.24)	0.883
	IG	58.3	70.1	57.8				
I can share my feelings/emotions with my professionals *%*	CAU	58.7	45.6	54.9	**2.46 (1.07 to 5.65)**	**0.035**	1.88 (0.84 to 4.21)	0.125
	IG	57.4	65.2	65.8				

Statistics based on the imputed dataset. N of alcohol is 84 (only people who drank were analyzed), all other *n* = 147. Numbers in bold indicate significant results

*B* parameter estimate of being in the IG for the difference *T*1-*T*0 or *T*2-*T*0, *CI* confidence interval, *SD* standard deviation, *CAU* care-as-usual, *IG* intervention group, *OR* odds ratio for being in the IG compared to CAU regarding the improvement in health behaviors between baseline and T1 or T2

^**1**^(PAM) Patient Activation Measure: scores from 0 to 100 with higher scores representing better patient activation

^**2**^Medication adherence: scores from 5 to 25 with higher scores representing better adherence

^**3**^Adequate physical activity: at least 150 min of exercise per week

^**4**^AAHLS questionnaire: scores from 10 to 30 with higher scores representing better health literacy

^**5**^eGFR = estimated glomerular filtration rate in ml/min/1.73m^2^; results of *T*1 not reported because they were not reliable (missing not at random); intervention effect *T*0-*T*2 is not corrected for eGFR

### Health care professional outcomes

The intervention effectively improved the self-reported use of health literacy communication strategies at *T*1 (*B* = 0.64, 95%CI [0.33, 0.95]) and *T*2 (*B* = 0.56, 95%CI [0.19, 0.93]). It enhanced the use of strategies in gathering information, providing information, shared decision-making, and enabling self-management (see Table 3).

**Table 3:** Linear regression of primary and secondary professional outcomes for the change between T0-T1 and T0-T2

Outcomes	Gr	*T*0	*n*	*T*1	*n*	*T*2	*n*	Intervention effect *T*0-*T*1 B (95% CI) *p*		Intervention effect *T*0-*T*2 B (95% CI) *p*	
HL knowledge* mean (SD)	CAU	5.25 (0.86)	18	5.19 (0.68)	18	5.14 (0.59)	18	**0.49 (0.04–0.93)**	**0.034**	**0.66 (0.31–1.01)**	** < 0.001**
	IG	4.37 (0.88)	30	5.47 (0.59)	27	5.65 (0.46)	28				
Total use of HL strategies mean (SD)	CAU	4.48 (0.55)	18	4.54 (0.69)	18	4.64 (0.52)	18	**0.64 (0.33–0.95)**	** < 0.001**	**0.56 (0.19–0.93)**	**0.004**
	IG	4.15 (0.57)	30	4.89 (0.52)	27	5.03 (0.67)	28				
Use of health literacy communication strategies within separate domains:											
Gathering information mean (SD)	CAU	5.22 (0.52)	18	5.31 (0.77)	18	5.44 (0.55)	18	**0.43 (0.04–0.81)**	**0.031**	0.17 (-0.22–0.57)	0.385
	IG	4.90 (0.66)	30	5.56 (0.55)	27	5.54 (0.68)	28				
Providing information mean (SD)	CAU	4.60 (0.70)	18	4.64 (0.79)	18	4.77 (0.80)	18	**0.38 (0.04–0.73)**	**0.030**	0.40 (-0.05–0.83)	0.077
	IG	4.26 (0.67)	30	4.90 (0.41)	27	5.08 (0.67)	28				
Shared decision-making mean (SD)	CAU	4.49 (0.81)	18	4.44 (0.98)	18	4.38 (0.67)	18	**0.80 (0.36–1.24)**	** < 0.001**	**0.93 (0.47–1.39)**	** < 0.001**
	IG	3.94 (0.84)	30	4.74 (0.90)	27	4.88 (0.97)	28				
Enabling self-management mean (SD)	CAU	3.79 (0.91)	18	3.89 (0.83)	18	4.00 (0.72)	18	**0.79 (0.40–1.18)**	** < 0.001**	**0.83 (0.38–1.27)**	** < 0.001**
	IG	3.55 (0.73)	30	4.39 (0.78)	27	4.63 (0.87)	28				

Numbers in bold indicate significant results

*Gr* group, *n* number of participants included in the analysis, *B* parameter estimate of being in the IG for the difference *T*1-*T*0 or *T*2-*T*0, *CI* confidence interval, *HL* health literacy, *SD* standard deviation, *CAU* care-as-usual, *IG* = intervention group

^*^At *T*0, there was a significant difference for health literacy knowledge between the IG and CAU (*p* = 0.001)

### Sensitivity and subgroup analyses

The results with the non-imputed dataset and without correction for eGFR were similar to the main analysis (Supplementary files 3 and 4). Subgroup analyses revealed some differences (Supplementary files 5, 6, 7 and 8). For patients at risk, at T2, the intervention tended to positively affect physical activity and fluid intake. For patients with limited health literacy, the intervention effect on hypertension and self-management was similar, but on consultation, outcomes were weaker. The intervention was more effective in nephrology clinics. For patients who used ≥2 intervention components, effectiveness was higher for consultation outcomes.

### Process evaluation

Overall, patients were satisfied. They rated GoYK with 7.75 out of 10 and 97% of the patients considered GoYK comprehensible. Seventy-five percent used two or three components. Optimal and suboptimal users did not differ in background characteristics. Patients with limited health literacy were more satisfied with the website and brochures. Patients with adequate health literacy used the consultation card more. Satisfaction, usefulness, and use of the intervention were lower among patients from general practices compared to nephrology clinics. Professionals graded the intervention with 8.17 out of 10 and expressed confidence in using health literacy strategies in the future.

## Discussion

GoYK did not affect patients’ self-management of health behaviors. However, it reduced hypertension and improved the use of health literacy strategies by professionals and the consultation quality. Patients and professionals considered the intervention useful, though it was less suitable for patients with limited health literacy and from general practices.

The intervention had no significant effect on self-management or patient activation. Behavioral interventions often show no effect [20]. Some reasons could explain our results. First, patients, in general, showed good lifestyles and patient activation at baseline, which probably induced a ceiling effect. This finding might seem incompatible, considering our focus on patients with limited health literacy. However, many patients have experienced CKD for years, and we expect this to be the result of years of treatment. Second, another study found that lower patient activation is associated with having a lower eGFR [21]. Potentially, the more complex healthcare needs of many patients in our sample make improvements in activation and self-management difficult. Third, the use of self-reported measures has limitations, and objective measures might show lifestyle improvements better [22].

The decrease in hypertension in the intervention group is promising for preventing kidney function deterioration [23]. GoYK appears to be the first CKD health literacy intervention affecting a clinical parameter [14]. We hypothesize the intervention induced a variety of lifestyle improvements in patients with inadequate health behaviors, leading to these better blood pressures. Our study then adds evidence showing that intervening on lifestyle can improve hypertension [24]. The Causal Pathway model of Paasche-Orlow and Wolf helps to understand the mechanisms behind this finding. The model theorizes that health literacy influences self-management and patient-professional communication, resulting in the generally worse health outcomes of patients with limited health literacy [25]. Following this model, improved communication may have contributed to the reduction in hypertension [26].

This effect of communication is plausible, as GoYK improved the use of health literacy strategies by professionals and the perceived quality of consultations. This confirms the usefulness of training professionals on health literacy [16, 21], to which the unique strategies of GoYK potentially contributed. GoYK builds the competences of patients and professionals, and guides the discussion of important health literacy-related barriers. Thereby, the specific needs of these patients [27] are, expectedly, better integrated in consultations. However, GoYK did not meet all patients’ needs: the effect on consultation-related outcomes was lower for patients with limited health literacy. Perhaps these patients might benefit more from simpler interventions targeting one problem at a time [28].

We noticed that patients from general practices considered the intervention less useful. CKD is often not explained well by general practitioners [5]. Improving disease awareness is a precondition to engaging in self-management, even more so in patients with limited health literacy [29]. GoYK could have targeted this more, to create a sense of urgency, before addressing self-management. In addition, we noticed 25% of the patients utilized the intervention sub-optimally. To optimize use, the intervention needs better integration in the organizations’ care processes. For example, we suggest the card be sent with a consultation invite, or that the dietitian provides our developed lifestyle information.

Study strengths are the thorough study design including multiple organizations responsible for primary and secondary CKD care, the use of validated and objective measures, and a process evaluation. The work also has limitations. First, in quasi-experimental studies, there is a performance bias risk. Treatment policies within different organizations potentially influenced our results. Second, we did not find an effect on our primary outcomes, which may suggest other routes by which the intervention leads to reduction of hypertension than we hypothesized originally. Third, people with very low health literacy seem underrepresented, because consent procedures might preclude people facing difficulties with reading or language proficiency from participating, for example when they need to read information letters.

Our study holds implications for research. Our intervention [15] is a blueprint for researchers aiming to optimize self-management and consultations in many care settings and contexts. We recommend the development and testing of more interventions for patients in general practices, as GoYK was less effective for them. At the same time, we suggest replication of our work to better understand how the intervention works exactly. In addition, considering the participation rate and underrepresentation of patients with very low health literacy, we suggest additional intervention research to evaluate the acceptance and effectiveness of the intervention in the most vulnerable groups.

Our study has implications for practice. Our intervention GoYK contains useful components to optimize CKD care for patients with limited health literacy. We suggest care organizations apply similar strategies to optimize patients’ health, self-management and consultations. Second, we advise incorporation of evidence-based health literacy training, like ours, in the agenda of healthcare studies and educational programs of care organizations.

In conclusion, we showed that our intervention, targeting CKD patients and professionals simultaneously, improved hypertension control and adherence to consultations. However, we did not find significant effects on self-management and patient activation. We suggest that further efforts should be addressed to the development and testing of educational  interventions, and more research to unravel by which mechanisms these interventions exert a clinically relevant effect.

## Supplementary Information

Below is the link to the electronic supplementary material.

Supplementary file1 (DOCX 102 kb)

## Data Availability

The datasets generated and/or analyzed during the current study are not publicly available due to privacy regulations in the Netherlands, but are available from the corresponding author on reasonable request.
