# Supplementary material for: Effectiveness of a health literacy intervention targeting both chronic kidney disease patients and health care professionals in primary and secondary care: a quasi-experimental study
Source: J Nephrol. 2024 Oct 4;37(9):2621–33. doi: 10.1007/s40620-024-02058-8 (PMC11663823; doi:10.1007/s40620-024-02058-8)
Supplement: Supplementary file 1 — Supplementary file1 (DOCX 102 kb) [file 40620_2024_2058_MOESM1_ESM.docx]

Supplementary files

[Supplementary file 1: The components of the intervention Grip on Your Kidneys 2](#_Toc169789206)

[Supplementary file 2: References on which the primary and secondary outcomes in this study are based 3](#_Toc169789207)

[Supplementary file 2: Crude analyses 4](#_Toc169789208)

[Supplementary file 3: Analyses without correction for eGFR for the imputed dataset 6](#_Toc169789209)

[Supplementary file 4: At risk analyses for dichotomous outcomes 8](#_Toc169789210)

[Supplementary file 5: Analyses for patients who used two or more intervention components, for the imputed dataset 9](#_Toc169789211)

[Supplementary file 6: Analyses for patients with limited health literacy for the imputed dataset 11](#_Toc169789212)

[Supplementary file 7: Analyses for general practices and hospitals separately for the imputed dataset 13](#_Toc169789213)

**Supplementary file 1: The components of the intervention Grip on Your Kidneys**
***Patient intervention***The patient intervention consisted of three components, with separate goals:

1. A website with simple text and videos with voice-over, or a brochure with simple text and picture stories. This component aimed to improve patients’ knowledge on CKD, self-management, and to teach how to actively participate in consultations with HCPs.
2. A consultation card with icons reflecting CKD-related symptoms, self-management, and barriers related to living with CKD and health literacy. This component aimed to support patients to prepare for the consultation. Additionally, the card supported patients and HCPs during the consultation by discussing the topics above and making shared decisions regarding self-management.
3. Seven topic-based brochures combined with motivational postcards. This component aimed to support patients to better incorporate self-management changes into daily life, overcome self-management barriers, and to ask for help if needed. The postcards aimed to remind patients of the different aspects of the intervention, motivate to start changing, and to compliment on achievements reached.

Patients received all intervention materials at once at their home address. Patients used GoYK independently and were invited to use all components. These took about 2.5 hours in total. The first step was to use the website or brochure. The second step was to use the card when they had a consultation with the HCP. Throughout the project, patients could use the seven brochures for topics that mattered to them. In the period between 3 and 9 months after starting the intervention, they received postcards every 1.5 months to remind and motivate them. Patients could decide to skip components that were not useful to them.  ***Professional intervention***
The professional intervention consisted of a 45-minutes e-learning and a 4-hour face-to-face workshop. The e-learning could be followed at home or at work, and provided a brief overview of health literacy, kidney disease and health literacy communication strategies, as a preparation for the workshop. The workshop took place in the hospital or general practice. It included various activities, such as oral presentations, videos, analysis of consultations, and case discussions. These activities enabled professionals to recognize patients with LHL, gather and provide information**,** promote shared decision-making**,** promote self-management, and motivate patients to improve and maintain self-management.

## **Supplementary file 2: References on which the primary and secondary outcomes in this study are based**

1. De Grauw W, De Leest K, Schenk P, et al (2014) NHG-standaard chronische nierschade. [https://richtlijnen.nhg.org/standaarden/chronische-nierschade. Accessed 24 Aug 2022](https://richtlijnen.nhg.org/standaarden/chronische-nierschade.%20Accessed%2024%20Aug%202022).
2. Humalda JK, Klaassen G, de Vries H, et al (2020) A Self-management Approach for Dietary Sodium Restriction in Patients With CKD: A Randomized Controlled Trial. Am J Kidney Dis 75:847–856. <https://doi.org/10.1053/j.ajkd.2019.10.012>
3. O’Halloran P, Kingsley M, Nicholson M, et al (2020) Responsiveness of the single item measure to detect change in physical activity. PLoS One 15:e0234420. <https://doi.org/10.1371/journal.pone.0234420>
4. De Grauw W, De Leest K, Schenk P, et al (2014) NHG-standaard chronische nierschade. <https://richtlijnen.nhg.org/standaarden/chronische-nierschade>. Accessed 24 Aug 2022
5. Dietisten Nierziekten Nederland (2019) Dieet bij chronische nierschade. [https://dietistennierziekten.nl/download/chronisch-nierschade-dieetrichtlijn. Accessed 7 Apr 2023](https://dietistennierziekten.nl/download/chronisch-nierschade-dieetrichtlijn.%20Accessed%207%20Apr%202023)
6. Chan AHY, Horne R, Hankins M, Chisari C (2020) The Medication Adherence Report Scale: A measurement tool for eliciting patients’ reports of nonadherence. Br J Clin Pharmacol 86:1281–1288. <https://doi.org/10.1111/bcp.14193>
7. Chinn D, McCarthy C (2013) All Aspects of Health Literacy Scale (AAHLS): Developing a tool to measure functional, communicative and critical health literacy in primary healthcare settings. Patient Educ Couns 90:247–253. <https://doi.org/10.1016/j.pec.2012.10.019>
8. de Haes H, Bensing J (2009) Endpoints in medical communication research, proposing a framework of functions and outcomes. Patient Educ Couns 74:287–294. <https://doi.org/10.1016/j.pec.2008.12.006>
9. Kaper MS, Sixsmith J, Koot JAR, et al (2018) Developing and pilot testing a comprehensive health literacy communication training for health professionals in three European countries. Patient Educ Couns 101:152–158. <https://doi.org/10.1016/j.pec.2017.07.017>
10. Study in NL. Dutch grading system. [https://www.studyinnl.org/dutch-education/dutch-grading-system. Accessed 3 Jan 2023](https://www.studyinnl.org/dutch-education/dutch-grading-system.%20Accessed%203%20Jan%202023)
11. Trimbos Institute (2022) Cijfers roken. https://www.trimbos.nl/kennis/cijfers/roken/. Accessed 5 Apr 2023
12. Trimbos Institute, Expertise Center Alcohol (2022) Cijfers alcoholgebruik jongeren. https://www.trimbos.nl/kennis/alcohol-in-cijfers/cijfers-alcoholgebruik-volwassenen/. Accessed 5 Apr 2023

## [**Supplementary file 2: Crude analyses**](#_Supplementary_file_1:)

| **Linear and logistic regressions of primary outcomes for the change between T0-T1 and T0-T2, without imputation and without correction for eGFR.** | | | | | | | | | | |
| --- | --- | --- | --- | --- | --- | --- | --- | --- | --- | --- |
| **Continuous primary outcomes** | **Group** | **Baseline** | **T1** | **T2** | **Intervention effect T0-T1** | | | **Intervention effect T0-T2** | | |
|  |  |  |  |  | **B (95% CI)** | **p** | **n** | **B (95% CI)** | **p** | **n** |
| PAM^1^ *mean (SD)* | CAU | 55.7 (10.0) | 56.5 (12.8) | 58.8 (14.0) | 1.65 (-2.06 to 5.37) | 0.380 | 125 | -2.25 (-6.64 to 2.13) | 0.311 | 121 |
|  | IG | 61.3 (13.3) | 63.7 (15.3) | 62.8 (12.5) |  |  |  |  |  |  |
| Medication adherence^2^ *mean (SD)* | CAU | 23.7 (2.0) | 23.9 (2.1) | 23.8 (1.7) | 0.19 (-0.29 to 0.68) | 0.429 | 134 | 0.35 (-0.24 to 0.95) | 0.240 | 127 |
|  | IG | 23.4 (2.2) | 23.5 (2.2) | 23.6 (2.0) |  |  |  |  |  |  |
| Alcohol (doses per week) *mean (SD)* | CAU | 6.3 (5.6) | 5.7 (5.0) | 6.3 (6.4) | 0.27 (-1.15 to 1.69) | 0.705 | 84 | -0.81 (-2.57 to 0.94) | 0.358 | 84 |
|  | IG | 4.7 (6.7) | 4.3 (5.2) | 3.8 (5.2) |  |  |  |  |  |  |
| Salt intake (days per week) *mean (SD)* | CAU | 5.1 (1.9) | 4.8 (2.1) | 5.2 (2.0) | -0.18 (-0.71 to 0.35) | 0.507 | 135 | -0.48 (-1.02 to 0.06) | 0.079 | 127 |
|  | IG | 4.9 (2.0) | 4.6 (1.9) | 4.5 (1.9) |  |  |  |  |  |  |
| **Dichotomous primary outcomes** | **Group** | **Baseline** | **T1** | **T2** | **OR (95% CI)** | **p** | **n** | **OR (95% CI)** | **p** | **n** |
| Adequate physical activity^3^ *%* | CAU | 43.9 | 38.1 | 43.1 | 2.04 (0.84 to 4.96) | 0.114 | 133 | 0.93 (0.41 to 2.13) | 0.865 | 125 |
|  | IG | 33.8 | 43.7 | 35.3 |  |  |  |  |  |  |
| Drink more than 1.5L a day *%* | CAU | 63.1 | 60.9 | 67.2 | 1.30 (0.58 to 2.89) | 0.529 | 133 | 0.70 (0.31 to 1.55) | 0.375 | 124 |
|  | IG | 47.5 | 59.2 | 54.4 |  |  |  |  |  |  |
| ***B*** *= parameter estimate of being in the IG for the difference T1-T0 or T2-T0,* ***CI*** *= confidence interval,* ***SD*** *= standard deviation,* ***CAU*** *= care-as-usual,* ***IG*** *= intervention group,* ***OR*** *odds ratio for improvement in the IG compared to CAU between baseline and T1 or T2.* ***^1^*** *Patient Activation Measure: scores from 0 to 100 with higher scores representing better patient activation.* ***^2^*** *Medication adherence: scores from 5 to 25 with higher scores representing better adherence.* ***^3^*** *Adequate physical activity: at least 150 minutes of exercise per week.* | | | | | | | | | | |

| **Linear and logistic regressions of secondary outcomes for the change between T0-T1 and T0-T2, without imputation and without correction for eGFR.** | | | | | | | | | | |
| --- | --- | --- | --- | --- | --- | --- | --- | --- | --- | --- |
| **Continuous secondary outcomes** | **Group** | **Baseline** | **T1** | **T2** | **Intervention effect T0-T1** | | | **Intervention effect T0-T2** | | |
|  |  |  |  |  | **B (95% CI)** | **p** | **n** | **B (95% CI)** | **p** | **n** |
| Health literacy^1^ *mean (SD)* | CAU | 23.0 (3.1) | 23.8 (3.0) | 23.7 (3.1) | -0.27 (-1.18 to 0.63) | 0.552 | 135 | -0.19 (-1.06 to 0.67) | 0.656 | 126 |
|  | IG | 23.9 (3.1) | 24.1 (2.7) | 24.2 (3.0) |  |  |  |  |  |  |
| N of lifestyle topics discussed in  consultation *mean (SD)* | CAU | 1.5 (1.1) | 1.0 (1.1) | 1.2 (1.2) | **0.84 (0.39 to 1.29)** | **<0.001** | **135** | **0.78 (0.31 to 1.26)** | **0.001** | **127** |
|  | IG | 1.0 (1.3) | 1.3 (1.3) | 1.4 (1.4) |  |  |  |  |  |  |
| eGFR^2^ *mean (SD)* | CAU | 33.3 (14.5) |  | 32.1 (16.9) |  | | | -1.14 (-3.12 to 0.83) | 0.253 | 114 |
|  | IG | 43.0 (15.8) |  | 41.0 (17.3) |  |  |  |  |  |  |
| BMI *mean (SD)* | CAU | 28.1 (5.4) | 28.2 (5.4) | 27.8 (4.4) | 0.15 (-0.21 to 0.52) | 0.401 | 132 | -0.07 (-0.54 to 0.40) | 0.768 | 126 |
|  | IG | 27.5 (4.4) | 27.6 (4.6) | 27.5 (4.5) |  |  |  |  |  |  |
| **Dichotomous secondary outcomes** | **Group** | **Baseline** | **T1** | **T2** | **OR (95% CI)** | **p** | **n** | **OR (95% CI)** | **p** | **n** |
| Hypertension *%* | CAU | 46.0 | 53.5 | 59.6 | 0.43 (0.18 to 1.02) | 0.055 | 95 | **0.35 (0.16 to 0.78)** | **0.010** | **106** |
|  | IG | 38.2 | 30.2 | 32.7 |  |  |  |  |  |  |
| Feeling understood by HCPs *%* | CAU | 70.8 | 69.6 | 72.5 | **5.15 (1.54 to 17.27)** | **0.008** | **115** | 1.42 (0.58 to 3.47) | 0.444 | 111 |
|  | IG | 83.8 | 92.3 | 78.1 |  |  |  |  |  |  |
| Encouraged by HCPs to ask questions *%* | CAU | 53.8 | 47.3 | 60.8 | **2.48 (1.08 to 5.69)** | **0.033** | **114** | 1.13 (0.51 to 2.52) | 0.770 | 110 |
|  | IG | 66.7 | 68.2 | 62.5 |  |  |  |  |  |  |
| HCPs listen to my preferred approach *%* | CAU | 58.5 | 55.4 | 64.7 | **3.30 (1.34 to 8.14)** | **0.009** | **115** | 1.30 (0.58 to 2.93) | 0.526 | 109 |
|  | IG | 62.5 | 75.8 | 68.3 |  |  |  |  |  |  |
| I can share my feelings/emotions  with my HCPs *%* | CAU | 59.4 | 47.3 | 64.7 | **3.81 (1.67 to 8.72)** | **0.002** | **113** | 2.37 (0.93 to 6.06) | 0.071 | 109 |
|  | IG | 61.1 | 72.7 | 76.6 |  |  |  |  |  |  |
| *Numbers* ***in bold*** *indicate significant results.* ***B*** *= parameter estimate of being in the IG for the difference T1-T0 or T2-T0,* ***CI*** *= confidence interval,* ***SD*** *= standard deviation,* ***CAU*** *= care-as-usual,* ***IG*** *= intervention group,* ***OR*** *= odds ratio for improvement in the IG compared to CAU between baseline and T1 or T2. ^1^ AAHLS questionnaire: scores from 10 to 30 with higher scores representing better health literacy. ^2^ eGFR = estimated glomerular filtration rate in ml/min/1.73m^2^; results of T1 not reported because they were not reliable (missing not at random).* | | | | | | | | | | |

## **Supplementary file 3: Analyses without correction for eGFR for the imputed dataset**

| **Linear and logistic regressions of primary outcomes for the change between T0-T1 and T0-T2, with imputation and without correction for eGFR.** | | | | | | | | |
| --- | --- | --- | --- | --- | --- | --- | --- | --- |
| **Continuous primary outcomes** | **Group** | **Baseline** | **T1** | **T2** | **Intervention effect T0-T1** | | **Intervention effect T0-T2** | |
|  |  |  |  |  | **B (95% CI)** | **p** | **B (95% CI)** | **p** |
| PAM^1^ *mean (SD)* | CAU | 55.6 (10.0) | 56.3 (14.2) | 59.0 (13.1) | 0.76 (-4.84 to 6.37) | 0.789 | -2.63 (-6.91 to 1.64) | 0.227 |
|  | IG | 61.7 (13.6) | 63.1 (17.5) | 62.5 (11.4) |  |  |  |  |
| Medication adherence^2^ *mean (SD)* | CAU | 23.8 (2.0) | 23.7 (2.5) | 23.6 (2.1) | -0.15 (-1.15 to 0.85) | 0.770 | 0.01 (-0.93 to 0.94) | 0.988 |
|  | IG | 23.4 (2.2) | 23.2 (2.9) | 23.2 (2.6) |  |  |  |  |
| Alcohol (doses per week) *mean (SD)* | CAU | 6.4 (5.5) | 5.7 (5.0) | 6.3 (6.3) | 0.27 (-1.15 to 1.69) | 0.710 | -0.83 (-2.58 to 0.92) | 0.352 |
|  | IG | 4.6 (6.5) | 4.2 (5.1) | 3.7 (5.1) |  |  |  |  |
| Salt intake (days per week) *mean (SD)* | CAU | 5.1 (1.9) | 4.8 (2.1) | 5.1 (2.1) | -0.04 (-0.67 to 0.60) | 0.910 | -0.53 (-1.25 to 0.20) | 0.152 |
|  | IG | 4.9 (1.9) | 4.5 (2.0) | 4.4 (2.0) |  |  |  |  |
| **Dichotomous primary outcomes** | **Group** | **Baseline** | **T1** | **T2** | **OR (95% CI)** | **p** | **OR (95% CI)** | **p** |
| Adequate physical activity^3^ *%* | CAU | 44.1 | 37.5 | 42.4 | 1.82 (0.76 to 4.36) | 0.178 | 0.87 (0.40 to 1.87) | 0.719 |
|  | IG | 34.6 | 42.1 | 35.7 |  |  |  |  |
| Drink more than 1.5L a day *%* | CAU | 62.8 | 60.9 | 66.8 | 1.22 (0.56 to 2.66) | 0.622 | 0.77 (0.34 to 1.73) | 0.052 |
|  | IG | 48.0 | 58.3 | 56.4 |  |  |  |  |
| *Statistics based on the imputed dataset.* ***n*** *of alcohol is 84 (only people who drank were analysed), all other* ***n*** *= 147.* ***B*** *= parameter estimate of being in the IG for the difference T1-T0 or T2-T0,* ***CI*** *= confidence interval,* ***SD*** *= standard deviation,* ***CAU*** *= care-as-usual,* ***IG*** *= intervention group,* ***OR*** *= odds ratio for improvement in the IG compared to CAU between baseline and T1 or T2.* ***^1^*** *Patient Activation Measure: scores from 0 to 100 with higher scores representing better patient activation.* ***^2^*** *Medication adherence: scores from 5 to 25 with higher scores representing better adherence.* ***^3^*** *Adequate physical activity: at least 150 minutes of exercise per week.* | | | | | | | | |

| **Linear and logistic regressions of secondary outcomes for the change between T0-T1 and T0-T2, with imputation and without correction for eGFR.** | | | | | | | | |
| --- | --- | --- | --- | --- | --- | --- | --- | --- |
| **Continuous secondary outcomes** | **Group** | **Baseline** | **T1** | **T2** | **Intervention effect T0-T1** | | **Intervention effect T0-T2** | |
|  |  |  |  |  | **B (95% CI)** | **p** | **B (95% CI)** | **p** |
| Health literacy^1^ *mean (SD)* | CAU | 23.0 (3.1) | 23.7 (3.2) | 23.6 (3.3) | -0.55 (-1.70 to 0.60) | 0.348 | -0.40 (-1.62 to 0.82) | 0.515 |
|  | IG | 23.8 (3.1) | 24.0 (3.1) | 24.0 (3.4) |  |  |  |  |
| N of lifestyle topics discussed in  consultation *mean (SD)* | CAU | 1.5 (1.1) | 1.0 (1.2) | 1.3 (1.3) | **0.87 (0.37 to 1.38)** | **0.001** | **0.71 (0.18 to 1.24)** | **0.009** |
|  | IG | 1.1 (1.3) | 1.4 (1.4) | 1.5 (1.5) |  |  |  |  |
| eGFR^2^ *mean (SD)* | CAU | 33.9 (14.9) |  | 32.7 (15.0) |  | | -2.39 (-6.01 to 1.23) | 0.195 |
|  | IG | 43.3 (16.2) |  | 39.7 (15.4) |  |  |  |  |
| BMI *mean (SD)* | CAU | 28.1 (5.4) | 28.3 (5.6) | 27.9 (4.9) | 0.27 (-1.27 to 1.81) | 0.730 | 0.37 (-1.17 to 1.90) | 0.637 |
|  | IG | 27.5 (4.4) | 27.9 (5.9) | 27.6 (5.1) |  |  |  |  |
| **Dichotomous secondary outcomes** | **Group** | **Baseline** | **T1** | **T2** | **OR (95% CI)** | **p** | **OR (95% CI)** | **p** |
| Hypertension *%* | CAU | 46.9 | 44.2 | 50.4 | 0.53 (0.23 to 1.22) | 0.134 | **0.38 (0.18 to 0.82)** | **0.014** |
|  | IG | 42.1 | 29.3 | 27.9 |  |  |  |  |
| Feeling understood by HCPs *%* | CAU | 70.5 | 63.5 | 61.7 | 2.35 (0.88 to 6.28) | 0.089 | 1.21 (0.58 to 2.50) | 0.614 |
|  | IG | 79.9 | 80.7 | 67.0 |  |  |  |  |
| Encouraged by HCPs to ask questions *%* | CAU | 53.6 | 44.9 | 52.4 | 1.98 (0.89 to 4.42) | 0.094 | 1.07 (0.51 to 2.26) | 0.863 |
|  | IG | 61.4 | 62.3 | 55.7 |  |  |  |  |
| HCPs listen to my preferred approach *%* | CAU | 58.3 | 52.7 | 55.6 | 2.39 (0.99 to 5.77) | 0.052 | 1.10 (0.54 to 2.25) | 0.797 |
|  | IG | 58.3 | 70.1 | 57.8 |  |  |  |  |
| I can share my feelings/emotions  with my HCPs *%* | CAU | 58.7 | 45.6 | 54.9 | **2.36 (1.08 to 5.15)** | **0.032** | 1.69 (0.78 to 3.66) | 0.186 |
|  | IG | 57.4 | 65.2 | 65.8 |  |  |  |  |
| *Statistics based on the imputed dataset. Numbers* ***in bold*** *indicate significant results.* ***n*** *of all analyses = 147.* ***B*** *= parameter estimate of being in the IG for the difference T1-T0 or T2-T0,* ***CI*** *= confidence interval,* ***SD*** *= standard deviation,* ***CAU*** *= care-as-usual,* ***IG*** *= intervention group,* ***OR*** *= odds ratio for improvement in the IG compared to CAU between baseline and T1 or T2. ^1^ AAHLS questionnaire: scores from 10 to 30 with higher scores representing better health literacy. ^2^ eGFR = estimated glomerular filtration rate in ml/min/1.73m^2^; results of T1 not reported because they were not reliable (missing not at random).* | | | | | | | | |

## [**Supplementary file 4: At**](#_Supplementary_file_1:) **risk analyses for dichotomous outcomes**

| **Logistic regressions of dichotomous primary and secondary outcomes, for patients at risk, for the change between T0-T1 and T0-T2** | | | | | | | | |
| --- | --- | --- | --- | --- | --- | --- | --- | --- |
| **Dichotomous primary outcomes** | **Group** | **Baseline** | **T1** | **T2** | **OR (95% CI)** | **p** | **OR (95% CI)** | **p** |
| Adequate physical activity^1^ *%* | CAU | 44.1 | 37.5 | 42.4 | 3.40 (0.85 to 13.64) | 0.085 | 1.98 (0.60 to 6.48) | 0.257 |
|  | IG | 34.6 | 42.1 | 35.7 |  |  |  |  |
| Fluid intake of ≥1.5L a day *%* | CAU | 62.8 | 60.9 | 66.8 | 2.66 (0.79 to 8.96) | 0.115 | 1.38 (0.43 to 4.41) | 0.587 |
|  | IG | 48.0 | 58.3 | 56.4 |  |  |  |  |
| **Dichotomous secondary outcomes** | **Group** | **Baseline** | **T1** | **T2** | **OR (95% CI)** | **p** | **OR (95% CI)** | **p** |
| Hypertension *%* | CAU | 46.9 | 44.2 | 50.4 | 2.20 (0.66 to 7.34) | 0.199 | **3.85 (1.08 to 13.68)** | **0.038** |
|  | IG | 42.1 | 29.3 | 27.9 |  |  |  |  |
| Feeling understood by HCPs *%* | CAU | 70.5 | 63.5 | 61.7 | 3.24 (0.62 to 16.68) | 0.165 | 1.53 (0.31 to 7.43) | 0.598 |
|  | IG | 79.9 | 80.7 | 67.0 |  |  |  |  |
| Encouraged by HCPs to ask questions *%* | CAU | 53.6 | 44.9 | 52.4 | 2.42 (0.67 to 8.84) | 0.180 | 0.73 (0.22 to 2.36) | 0.596 |
|  | IG | 61.4 | 62.3 | 55.7 |  |  |  |  |
| HCPs listen to my preferred approach *%* | CAU | 58.3 | 52.7 | 55.6 | 2.41 (0.68 to 8.49) | 0.171 | 1.15 (0.36 to 3.69) | 0.819 |
|  | IG | 58.3 | 70.1 | 57.8 |  |  |  |  |
| I can share my feelings/emotions with my HCPs *%* | CAU | 58.7 | 45.6 | 54.9 | 3.47 (0.96 to 12.57) | 0.059 | 1.91 (0.58 to 6.30) | 0.289 |
|  | IG | 57.4 | 65.2 | 65.8 |  |  |  |  |
| *Statistics based on the imputed dataset. Numbers* ***in bold*** *indicate significant results.* ***n*** *of all analyses = 147,* ***CI*** *= confidence interval,* ***CAU*** *= care-as-usual,* ***IG*** *= intervention group,* ***OR*** *= odds ratio for improvement in the IG compared to CAU between baseline and T1 or T2; only participants at risk were analysed.* ***^1^*** *Adequate physical activity: at least 150 minutes of exercise per week.* | | | | | | | | |

## **Supplementary file 5: Analyses for patients who used two or more intervention components, for the imputed dataset**

| **Linear and logistic regressions of primary outcomes for the change between T0-T1 and T0-T2, with imputation and correction for eGFR.** | | | | | | | | |
| --- | --- | --- | --- | --- | --- | --- | --- | --- |
| **Continuous primary outcomes** | **Group** | **Baseline** | **T1** | **T2** | **Intervention effect T0-T1** | | **Intervention effect T0-T2** | |
|  |  |  |  |  | **B (95% CI)** | **p** | **B (95% CI)** | **p** |
| PAM^1^ *mean (SD)* | CAU | 55.6 (10.0) | 56.3 (14.2) | 59.0 (13.1) | 1.48 (-3.50 to 6.46) | 0.560 | **-4.58 (-8.99 to -0.17)** | **0.042** |
|  | IG | 62.1 (12.8) | 64.8 (15.2) | 62.3 (11.1) |  |  |  |  |
| Medication adherence^2^ *mean (SD)* | CAU | 23.8 (2.0) | 23.7 (2.5) | 23.6 (2.1) | 0.12 (-0.70 to 0.94) | 0.773 | 0.21 (-0.64 to 1.06) | 0.626 |
|  | IG | 23.4 (2.3) | 23.4 (2.4) | 23.5 (2.3) |  |  |  |  |
| Alcohol (doses per week) *mean (SD)* | CAU | 6.4 (5.5) | 5.7 (5.0) | 6.3 (6.3) | 0.14 (-1.43 to 1.71) | 0.863 | -1.20 (-3.07 to 0.67) | 0.207 |
|  | IG | 4.3 (6.4) | 3.8 (4.4) | 3.3 (4.5) |  |  |  |  |
| Salt intake (days per week) *mean (SD)* | CAU | 5.1 (1.9) | 4.8 (2.1) | 5.1 (2.1) | -0.16 (-0.75 to 0.43) | 0.592 | **-0.71 (-1.41 to -0.01)** | **0.047** |
|  | IG | 5.1 (1.8) | 4.5 (1.9) | 4.4 (1.9) |  |  |  |  |
| **Dichotomous primary outcomes** | **Group** | **Baseline** | **T1** | **T2** | **OR (95% CI)** | **p** | **OR (95% CI)** | **p** |
| Adequate physical activity^3^ *%* | CAU | 44.1 | 37.5 | 42.4 | 1.75 (0.68 to 4.48) | 0.245 | 0.91 (0.39 to 2.13) | 0.830 |
|  | IG | 37.5 | 41.4 | 34.8 |  |  |  |  |
| Drink more than 1.5L a day *%* | CAU | 62.8 | 60.9 | 66.8 | 1.57 (0.65 to 1.57) | 0.319 | 0.81 (0.34 to 1.95) | 0.638 |
|  | IG | 49.9 | 61.1 | 54.0 |  |  |  |  |
| *Statistics based on the imputed dataset, but only participants who used ≥2 components of the intervention were included in the analysis. Numbers* ***in bold*** *indicate significant results.* ***n*** *of alcohol is 75 (only people who drank were analysed); all other* ***n*** *= 129.* ***B*** *= parameter estimate of being in the IG for the difference T1-T0 or T2-T0,* ***CI*** *= confidence interval,* ***SD*** *= standard deviation,* ***CAU*** *= care-as-usual,* ***IG*** *= intervention group,* ***OR*** *= odds ratio for improvement in the IG compared to CAU between baseline and T1 or T2.* ***^1^*** *Patient Activation Measure: scores from 0 to 100 with higher scores representing better patient activation.* ***^2^*** *Medication adherence: scores from 5 to 25 with higher scores representing better adherence.* ***^3^*** *Adequate physical activity: at least 150 minutes of exercise per week.* | | | | | | | | |

| **Linear and logistic regressions of secondary outcomes for the change between T0-T1 and T0-T2, with imputation and correction for eGFR.** | | | | | | | | |
| --- | --- | --- | --- | --- | --- | --- | --- | --- |
| **Continuous secondary outcomes** | **Group** | **Baseline** | **T1** | **T2** | **Intervention effect T0-T1** | | **Intervention effect T0-T2** | |
|  |  |  |  |  | **B (95% CI)** | **p** | **B (95% CI)** | **p** |
| Health literacy^1^ *mean (SD)* | C | 23.0 (3.1) | 23.7 (3.2) | 23.6 (3.3) | 0.03 (-1.03 to 1.08) | 0.963 | -0.07 (-1.23 to 1.09) | 0.905 |
|  | I | 23.6 (3.1) | 24.1 (2.7) | 24.1 (3.2) |  |  |  |  |
| N of lifestyle topics discussed in  consultation *mean (SD)* | C | 1.5 (1.1) | 1.0 (1.2) | 1.3 (1.3) | **0.83 (0.33 to 1.32)** | **0.001** | **0.67 (0.13 to 1.20)** | **0.015** |
|  | I | 1.0 (1.4) | 1.4 (1.3) | 1.5 (1.4) |  |  |  |  |
| eGFR^2^ *mean (SD)* | C | 33.9 (14.9) |  | 32.7 (15.0) |  | | -2.65 (-5.90 to 0.61) | 0.111 |
|  | I | 42.7 (14.6) |  | 38.8 (15.0) |  |  |  |  |
| BMI *mean (SD)* | C | 28.1 (5.4) | 28.3 (5.6) | 27.9 (4.9) | -0.06 (-1.38 to 1.26) | 0.928 | 0.07 (-1.16 to 1.29) | 0.917 |
|  | I | 27.9 (4.7) | 28.1 (5.0) | 27.9 (4.9) |  |  |  |  |
| **Dichotomous secondary outcomes** | **Group** | **Baseline** | **T1** | **T2** | **OR (95% CI)** | **p** | **OR (95% CI)** | **p** |
| Hypertension *%* | C | 46.9 | 44.2 | 50.4 | 0.53 (0.21 to 1.31) | 0.169 | 0.47 (0.21 to 1.10) | 0.080 |
|  | I | 37.8 | 27.5 | 28.7 |  |  |  |  |
| Feeling understood by HCPs *%* | C | 70.5 | 63.5 | 61.7 | **4.37 (1.49 to 12.81)** | **0.007** | 1.45 (0.63 to 3.34) | 0.383 |
|  | I | 71.9 | 87.1 | 70.7 |  |  |  |  |
| Encouraged by HCPs to ask questions *%* | C | 53.6 | 44.9 | 52.4 | **3.74 (1.42 to 9.84)** | **0.008** | 1.23 (0.52 to 2.91) | 0.639 |
|  | I | 61.0 | 69.2 | 60.0 |  |  |  |  |
| HCPs listen to my preferred approach *%* | C | 58.3 | 52.7 | 55.6 | **3.14 (1.21 to 8.11)** | **0.018** | 1.13 (0.49 to 2.58) | 0.775 |
|  | I | 60.2 | 74.3 | 61.1 |  |  |  |  |
| I can share my feelings/emotions  with my HCPs *%* | C | 58.7 | 45.6 | 54.9 | **2.89 (1.20 to 6.93)** | **0.018** | 2.13 (0.90 to 5.03) | 0.086 |
|  | I | 58.9 | 70.2 | 69.3 |  |  |  |  |
| *Statistics based on the imputed dataset, but only participants who used ≥2 components of the intervention were included in the analysis. Numbers* ***in bold*** *indicate significant results.* ***n*** *of all analyses = 129.* ***B*** *= parameter estimate of being in the IG for the difference T1-T0 or T2-T0,* ***CI*** *= confidence interval,* ***SD*** *= standard deviation,* ***CAU*** *= care-as-usual,* ***IG*** *= intervention group,* ***OR*** *= odds ratio for improvement in the IG compared to CAU between baseline and T1 or T2. ^1^ AAHLS questionnaire: scores from 10 to 30 with higher scores representing better health literacy. ^2^ eGFR = estimated glomerular filtration rate in ml/min/1.73m^2^; results of T1 not reported because they were not reliable (missing not at random); intervention effect T0-T2 is not corrected for eGFR.* | | | | | | | | |

## **Supplementary file 6: Analyses for patients with limited health literacy for the imputed dataset**

| **Linear and logistic regressions of primary outcomes for the change between T0-T1 and T0-T2, with imputation and correction for eGFR.** | | | | | | | | |
| --- | --- | --- | --- | --- | --- | --- | --- | --- |
| **Continuous primary outcomes** | **Group** | **Baseline** | **T1** | **T2** | **Intervention effect T0-T1** | | **Intervention effect T0-T2** | |
|  |  |  |  |  | **B (95% CI)** | **p** | **B (95% CI)** | **p** |
| PAM^1^ *mean (SD)* | C | 53.7 (9.2) | 54.2 (12.8) | 56.9 (12.2) | 1.04 (-5.49 to 7.57) | 0.754 | -0.75 (-5.76 to 4.27) | 0.771 |
|  | I | 58.8 (11.6) | 61.0 (15.9) | 62.3 (9.5) |  |  |  |  |
| Medication adherence^2^ *mean (SD)* | C | 24.1 (1.2) | 23.8 (2.0) | 23.6 (2.1) | 0.11 (-0.91 to 1.13) | 0.827 | 0.23 (-0.82 to 1.27) | 0.667 |
|  | I | 23.6 (2.0) | 23.4 (2.7) | 23.3 (2.5) |  |  |  |  |
| Alcohol (doses per week) *mean (SD)* | C | 5.9 (5.7) | 4.9 (4.5) | 6.0 (6.8) | -0.11 (-2.00 to 1.78) | 0.909 | -1.83 (-4.30 to 0.64) | 0.146 |
|  | I | 5.7 (7.7) | 4.7 (5.9) | 4.2 (5.9) |  |  |  |  |
| Salt intake (days per week) *mean (SD)* | C | 5.3 (1.8) | 4.9 (2.1) | 5.3 (2.0) | -0.14 (-0.87 to 0.59) | 0.704 | -0.74 (-1.65 to 0.17) | 0.109 |
|  | I | 5.2 (1.9) | 4.6 (2.0) | 4.4 (2.1) |  |  |  |  |
| **Dichotomous primary outcomes** | **Group** | **Baseline** | **T1** | **T2** | **OR (95% CI)** | **p** | **OR (95% CI)** | **p** |
| Adequate physical activity^3^ *%* | C | 36.1 | 31.0 | 36.2 | 1.93 (0.66 to 5.65) | 0.231 | 0.66 (0.24 to 1.85) | 0.432 |
|  | I | 40.0 | 43.1 | 30.3 |  |  |  |  |
| Drink more than 1.5L a day *%* | C | 61.0 | 58.5 | 61.7 | 1.20 (0.47 to 3.06) | 0.699 | 1.20 (0.46 to 3.16) | 0.710 |
|  | I | 44.4 | 53.3 | 58.2 |  |  |  |  |
| *Statistics based on the imputed dataset, but only participants with LHL were included (a score on the AAHLS ≤25). Numbers* ***in bold*** *indicate significant results.* ***n*** *of alcohol is 57 (only people who drank were analysed); all other* ***n*** *= 102.* ***B*** *= parameter estimate of being in the IG for the difference T1-T0 or T2-T0,* ***CI*** *= confidence interval,* ***SD*** *= standard deviation,* ***CAU*** *= care-as-usual,* ***IG*** *= intervention group,* ***OR*** *= odds ratio for improvement in the IG compared to CAU between baseline and T1 or T2. ^1^ Patient Activation Measure: scores from 0 to 100 with higher scores representing better patient activation. ^2^ Medication adherence: scores from 5 to 25 with higher scores representing better adherence. ^3^ Adequate physical activity: at least 150 minutes of exercise per week.* | | | | | | | | |

| **Linear and logistic regressions of secondary outcomes for the change between T0-T1 and T0-T2, with imputation and correction for eGFR.** | | | | | | | | |
| --- | --- | --- | --- | --- | --- | --- | --- | --- |
| **Continuous secondary outcomes** | **Group** | **Baseline** | **T1** | **T2** | **Intervention effect T0-T1** | | **Intervention effect T0-T2** | |
|  |  |  |  |  | **B (95% CI)** | **p** | **B (95% CI)** | **p** |
| Health literacy^1^ *mean (SD)* | C | 21.8 (2.4) | 22.6 (2.6) | 22.8 (3.1) | 0.17 (-1.21 to 1.56) | 0.805 | -0.10 (-1.58 to 1.38) | 0.893 |
|  | I | 22.1 (2.3) | 23.0 (2.9) | 22.9 (3.1) |  |  |  |  |
| N of lifestyle topics discussed in  consultation *mean (SD)* | C | 1.6 (1.1) | 1.1 (1.2) | 1.2 (1.3) | **0.74 (0.11 to 1.38)** | **0.021** | **0.82 (0.16 to 1.47)** | **0.015** |
|  | I | 1.2 (1.5) | 1.5 (1.4) | 1.7 (1.5) |  |  |  |  |
| eGFR^2^ *mean (SD)* | C | 34.8 (14.7) |  | 33.0 (15.3) |  | | -0.56 (-4.48 to 3.36) | 0.779 |
|  | I | 40.8 (15.1) |  | 38.4 (14.5) |  |  |  |  |
| BMI *mean (SD)* | C | 28.3 (5.4) | 28.5 (5.8) | 27.9 (4.7) | 0.05 (-1.98 to 2.09) | 0.960 | 0.24 (-1.45 to 1.93) | 0.776 |
|  | I | 27.1 (3.8) | 27.4 (5.2) | 27.0 (4.5) |  |  |  |  |
| **Dichotomous secondary outcomes** | **Group** | **Baseline** | **T1** | **T2** | **OR (95% CI)** | **p** | **OR (95% CI)** | **p** |
| Hypertension *%* | C | 50.7 | 38.2 | 43.5 | 0.93 (0.33 to 2.63) | 0.883 | 0.59 (0.23 to 1.51) | 0.268 |
|  | I | 44.1 | 32.3 | 29.0 |  |  |  |  |
| Feeling understood by HCPs *%* | C | 66.8 | 65.1 | 58.3 | 1.63 (0.55 to 4.85) | 0.380 | 1.18 (0.47 to 2.93) | 0.728 |
|  | I | 81.4 | 77.4 | 63.0 |  |  |  |  |
| Encouraged by HCPs to ask questions *%* | C | 48.6 | 42.9 | 50.0 | 2.08 (0.77 to 5.62) | 0.147 | 0.96 (0.38 to 2.41) | 0.933 |
|  | I | 60.4 | 58.5 | 52.3 |  |  |  |  |
| HCPs listen to my preferred approach *%* | C | 50.7 | 50.5 | 54.5 | 1.88 (0.71 to 4.98) | 0.207 | 0.96 (0.40 to 2.30) | 0.929 |
|  | I | 53.4 | 62.4 | 54.4 |  |  |  |  |
| I can share my feelings/emotions  with my HCPs *%* | C | 57.6 | 46.4 | 53.7 | 1.66 (0.65 to 4.26) | 0.291 | 1.73 (0.70 to 4.27) | 0.237 |
|  | I | 55.6 | 57.8 | 64.8 |  |  |  |  |
| *Statistics based on the imputed dataset, but only participants with LHL were included (a score on the AAHLS ≤25). Numbers* ***in bold*** *indicate significant results.* ***n*** *of all analyses = 102.* ***B*** *= parameter estimate of being in the IG for the difference T1-T0 or T2-T0,* ***CI*** *= confidence interval,* ***SD*** *= standard deviation,* ***CAU*** *= care-as-usual,* ***IG*** *= intervention group,* ***OR*** *odds ratio for improvement in the IG compared to CAU between baseline and T1 or T2. ^1^ AAHLS questionnaire: scores from 10 to 30 with higher scores representing better health literacy. ^2^ eGFR = estimated glomerular filtration rate in ml/min/1.73m^2^; results of T1 not reported because they were not reliable (missing not at random); intervention effect T0-T2 is not corrected for eGFR.* | | | | | | | | |

| **Supplementary file 7: Analyses for general practices and hospitals separately for the imputed dataset** | | | | | | | | |
| --- | --- | --- | --- | --- | --- | --- | --- | --- |
| **Linear and logistic regressions of primary outcomes for the change between T0-T1 and T0-T2, with imputation and correction for eGFR.** | | | | | | | | |
| **Continuous primary outcomes** | **Group** | **Baseline** | **T1** | **T2** | **Intervention effect T0-T1** | | **Intervention effect T0-T2** | |
|  |  |  |  |  | **B (95% CI)** | **p** | **B (95% CI)** | **p** |
| PAM^1^ *mean (SD) general practices* | C | 55.3 (7.7) | 57.0 (14.5) | 60.7 (12.0) | -2.00 (-13.58 to 9.59) | 0.734 | -4.52 (-11.87 to 2.84) | 0.228 |
|  | I | 61.3 (13.2) | 61.9 (18.7) | 63.4 (10.4) |  |  |  |  |
| PAM^1^ *mean (SD) hospitals* | C | 55.8 (11.3) | 55.8 (13.9) | 57.9 (13.7) | 1.57 (-5.25 to 8.38) | 0.651 | -3.24 (-8.93 to 2.44) | 0.264 |
|  | I | 61.9 (13.8) | 64.0 (16.5) | 61.9 (11.9) |  |  |  |  |
| Medication adherence^2^ *mean (SD) general practices* | C | 23.7 (1.7) | 23.8 (2.0) | 23.5 (2.0) | -0.49 (-2.55 to 1.58) | 0.641 | -0.41 (-1.95 to 1.14) | 0.603 |
|  | I | 23.8 (1.5) | 23.3 (3.2) | 23.2 (2.7) |  |  |  |  |
| Medication adherence^2^ *mean (SD) hospitals* | C | 23.8 (2.2) | 23.6 (2.8) | 23.6 (2.1) | 0.23 (-0.75 to 1.21) | 0.647 | -0.04 (-1.31 to 1.26) | 0.946 |
|  | I | 23.1 (2.5) | 23.1 (2.8) | 23.2 (2.6) |  |  |  |  |
| Alcohol (doses per week) *mean (SD) general practices* | C | 6.1 (4.6) | 5.3 (4.5) | 6.6 (5.7) | 0.64 (-0.76 to 2.03) | 0.370 | -1.70 (-4.08 to 0.68) | 0.162 |
|  | I | 4.5 (5.7) | 4.4 (5.3) | 3.5 (5.4) |  |  |  |  |
| Alcohol (doses per week) *mean (SD) hospitals* | C | 6.8 (6.6) | 6.2 (5.5) | 5.8 (7.0) | -0.29 (-3.17 to 2.59) | 0.844 | -0.58 (-3.66 to 2.50) | 0.711 |
|  | I | 4.7 (7.1) | 4.1 (4.9) | 3.8 (4.9) |  |  |  |  |
| Salt intake (days per week) *mean (SD) general practices* | C | 5.9 (1.7) | 5.6 (1.8) | 5.6 (1.8) | 0.02 (-1.09 to 1.12) | 0.977 | 0.13 (-1.14 to 1.39) | 0.841 |
|  | I | 5.0 (2.1) | 4.7 (2.1) | 4.8 (2.1) |  |  |  |  |
| Salt intake (days per week) *mean (SD) hospitals* | C | 4.6 (1.9) | 4.2 (2.2) | 4.8 (2.2) | -0.02 (-0.86 to 0.82) | 0.968 | **-1.15 (-2.21 to -0.10)** | **0.032** |
|  | I | 4.8 (1.8) | 4.4 (1.9) | 4.0 (1.9) |  |  |  |  |
| **Dichotomous primary outcomes** | **Group** | **Baseline** | **T1** | **T2** | **OR (95% CI)** | **p** | **OR (95% CI)** | **p** |
| Adequate physical activity^3^ *%,  general practices* | C | 44.9 | 38.7 | 43.2 | 2.57 (0.63 to 10.56) | 0.190 | 0.78 (0.21 to 3.00) | 0.721 |
|  | I | 40.7 | 49.6 | 36.6 |  |  |  |  |
| Adequate physical activity^3^ *%,  hospitals* | C | 43.6 | 36.7 | 41.8 | 1.80 (0.50 to 6.47) | 0.370 | 1.31 (0.43 to 3.94) | 0.633 |
|  | I | 30.5 | 37.0 | 35.0 |  |  |  |  |
| Drink more than 1.5L a day *% general practices* | C | 60.9 | 57.2 | 62.8 | 1.72 (0.37 to 8.11) | 0.492 | 1.04 (0.26 to 4.21) | 0.953 |
|  | I | 32.2 | 50.2 | 52.0 |  |  |  |  |
| Drink more than 1.5L a day *% hospitals* | C | 64.1 | 63.5 | 69.6 | 1.12 (0.38 to 3.29) | 0.837 | 0.88 (0.28 to 2.74) | 0.824 |
|  | I | 58.6 | 63.7 | 59.3 |  |  |  |  |
| *Statistics based on the imputed dataset. Numbers* ***in bold*** *indicate significant results.* ***n*** *of alcohol is 39 for GPs and 45 for hospitals (only people who drank were analysed); all other* ***n*** *= 59 for GPs and 88 for hospitals.* ***B*** *= parameter estimate of being in the IG for the difference T1-T0 or T2-T0,* ***CI*** *= confidence interval,* ***SD*** *= standard deviation,* ***CAU*** *= care-as-usual,* ***IG*** *= intervention group,* ***OR*** *= odds ratio for improvement in the IG compared to CAU between baseline and T1 or T2. ^1^ Patient Activation Measure: scores from 0 to 100 with higher scores representing better patient activation. ^2^ Medication adherence: scores from 5 to 25 with higher scores representing better adherence. ^3^ Adequate physical activity: at least 150 minutes of exercise per week.* | | | | | | | | |

| **Linear and logistic regressions of secondary outcomes for the change between T0-T1 and T0-T2, with imputation and correction for eGFR.** | | | | | | | | |
| --- | --- | --- | --- | --- | --- | --- | --- | --- |
| **Continuous secondary outcomes** | **Group** | **Baseline** | **T1** | **T2** | **Intervention effect T0-T1** | | **Intervention effect T0-T2** | |
|  |  |  |  |  | **B (95% CI)** | **p** | **B (95% CI)** | **p** |
| Health literacy^1^ *mean (SD)*  *general practices* | C | 23.4 (2.8) | 23.6 (3.6) | 24.0 (3.5) | -0.69 (-2.89 to 1.50) | 0.534 | -0.64 (-2.83 to 1.55) | 0.567 |
|  | I | 24.2 (2.8) | 23.7 (3.4) | 24.2 (3.4) |  |  |  |  |
| Health literacy^1^ *mean (SD)*  *Hospitals* | C | 22.7 (3.2) | 23.7 (2.8) | 23.4 (3.1) | -0.18 (-1.57 to 1.20) | 0.796 | 0.12 (-1.36 to 1.59) | 0.879 |
|  | I | 23.6 (3.3) | 24.1 (2.9) | 24.0 (3.3) |  |  |  |  |
| N of lifestyle topics discussed in  consultation *mean (SD), general practices* | C | 1.8 (0.9) | 1.2 (1.2) | 1.2 (1.2) | **1.01 (0.02 to 1.84)** | **0.017** | **1.05 (0.06 to 2.05)** | **0.039** |
|  | I | 0.7 (0.9) | 1.2 (1.3) | 1.3 (1.5) |  |  |  |  |
| N of lifestyle topics discussed in  consultation *mean (SD), hospitals* | C | 1.3 (1.2) | 0.9 (1.1) | 1.3 (1.4) | 0.55 (-0.14 to 1.23) | 0.118 | 0.41 (-0.29 to 1.10) | 0.250 |
|  | I | 1.3 (1.5) | 1.5 (1.4) | 1.6 (1.5) |  |  |  |  |
| eGFR^2^ *mean (SD)*  *general practices* | C | 47.0 (10.4) |  | 43.5 (10.9) |  | | -2.55 (-10.00 to 4.90) | 0.501 |
|  | I | 53.0 (12.8) |  | 47.0 (14.0) |  |  |  |  |
| eGFR^2^ *mean (SD)*  *hospitals* | C | 24.9 (10.1) |  | 25.3 (12.8) |  | | -2.32 (-5.56 to 0.91) | 0.159 |
|  | I | 36.8 (14.9) |  | 34.8 (14.3) |  |  |  |  |
| BMI *mean (SD)*  *general practices* | C | 26.6 (3.7) | 26.9 (4.5) | 26.5 (4.1) | 0.42 (0.78 to 2.58) | 0.277 | 0.24 (-2.41 to 2.88) | 0.859 |
|  | I | 26.7 (3.9) | 27.5 (6.5) | 27.0 (5.6) |  |  |  |  |
| BMI *mean (SD)*  *hospitals* | C | 29.2 (6.0) | 29.2 (6.1) | 28.7 (5.2) | 0.40 (-1.54 to 2.33) | 0.685 | 0.52 (-1.51 to 2.55) | 0.615 |
|  | I | 28.0 (4.6) | 28.2 (5.4) | 28.0 (4.7) |  |  |  |  |
| **Dichotomous secondary outcomes** | **Group** | **Baseline** | **T1** | **T2** | **OR (95% CI)** | **p** | **OR (95% CI)** | **p** |
| Hypertension *%*  *general practices* | C | 40.6 | 40.7 | 37.8 | 0.52 (0.19 to 2.25) | 0.376 | 0.79 (0.22 to 2.87) | 0.716 |
|  | I | 44.4 | 26.4 | 30.8 |  |  |  |  |
| Hypertension *%*  *hospitals* | C | 51.3 | 46.5 | 59.1 | 0.60 (0.19 to 1.86) | 0.374 | **0.32 (0.11 to 0.96)** | **0.043** |
|  | I | 40.6 | 31.3 | 25.9 |  |  |  |  |
| Feeling understood by HCPs *%*  *general practices* | C | 68.6 | 62.1 | 52.5 | 1.29 (0.32 to 5.23) | 0.725 | 1.14 (0.34 to 3.88) | 0.833 |
|  | I | 70.4 | 64.3 | 56.8 |  |  |  |  |
| Feeling understood by HCPs *%*  *hospitals* | C | 71.8 | 64.5 | 68.1 | n.a.^3^ |  | 1.10 (0.34 to 3.49) | 0.876 |
|  | I | 86.2 | 91.6 | 73.8 |  |  |  |  |
| Encouraged by HCPs to ask questions *%*  *general practices* | C | 49.5 | 44.1 | 47.5 | 1.18 (0.35 to 3.93) | 0.787 | 0.74 (0.22 to 2.53) | 0.627 |
|  | I | 50.1 | 46.8 | 42.6 |  |  |  |  |
| Encouraged by HCPs to ask questions *%*  *hospitals* | C | 56.4 | 45.5 | 55.8 | **4.24 (1.22 to 14.72)** | **0.023** | 0.95 (0.31 to 2.92) | 0.926 |
|  | I | 68.9 | 72.6 | 64.4 |  |  |  |  |
| HCPs listen to my preferred approach *%*  *general practices* | C | 60.9 | 57.2 | 50.8 | 1.17 (0.30 to 4.60) | 0.817 | 0.68 (0.20 to 2.28) | 0.526 |
|  | I | 48.7 | 55.7 | 45.2 |  |  |  |  |
| HCPs listen to my preferred approach *%*  *hospitals* | C | 56.4 | 49.6 | 58.8 | **4.79 (1.37 to 16.75)** | **0.014** | 0.86 (0.29 to 2.57) | 0.790 |
|  | I | 64.7 | 79.7 | 66.2 |  |  |  |  |
| I can share my feelings/emotions  with my HCPs *%, general practices* | C | 64.1 | 53.5 | 43.6 | 1.42 (0.38 to 5.38) | 0.603 | 1.70 (0.48 to 6.03) | 0.408 |
|  | I | 52.0 | 58.7 | 52.7 |  |  |  |  |
| I can share my feelings/emotions  with my HCPs *%, hospitals* | C | 55.0 | 40.3 | 62.7 | **4.11 (1.36 to 12.39)** | **0.012** | 1.38 (0.43 to 4.40) | 0.589 |
|  | I | 61.0 | 69.6 | 74.6 |  |  |  |  |
| *Statistics based on the imputed dataset. Numbers* ***in bold*** *indicate significant results.* ***n*** *of GP analyses = 59 and n of hospital analyses = 88.* ***B*** *= parameter estimate of being in the IG for the difference T1-T0 or T2-T0,* ***CI*** *= confidence interval,* ***SD*** *= standard deviation,* ***CAU*** *= care-as-usual,* ***IG*** *= intervention group,* ***OR*** *= odds ratio for improvement in the IG compared to CAU between baseline and T1 or T2. ^1^ AAHLS questionnaire: scores from 10 to 30 with higher scores representing better health literacy. ^2^ eGFR = estimated glomerular filtration rate in ml/min/1.73m^2^; results of T1 not reported because they were not reliable (missing not at random); intervention effect T0-T2 is not corrected for eGFR. ^3^ Unable to calculate due to too small n within answer categories.* | | | | | | | | |
